# V.A.D.'s Casualty Clearing Stations

**Published:** 1919-05-31

**Authors:** 


### V.A.D.'s Casualty Clearing Stations.

SIR,—We noticed in your issue of May 3 that Miss Alice Batt, V.A.D., was awarded the Albert Medal for gallantry in saving life. The account says that a fire broke out at No. 36 C.C.S., where Miss Batt was working in the theatre. Since she was handing instruments and needles to the operating surgeon we presume she was acting as theatre sister.

We think we are quite correct in stating that no V.A.D.s have ever been to C.C.S.'s except on pleasure trips, and even if they had been allowed to work at these advanced centres it would have been as nursing V.A.D.s and not as theatre sister—a highly responsible and difficult post. But putting aside the question of what might have been, we can clearly state that Miss Batt is *not* a V.A.D., but a trained sister, belonging either to the T.F.N.S. or Q.A.I.M.N.S.R.

We should be glad if you will confirm this and acknowledge the mistake in your next issue. There has been a good deal of heated discussion on the subject, and in view of the State Registration Bill and some of the proposed clauses it has not been without justification.

TWO OLD STAGERS.

May 17, 1919.

[The *London Gazette* of April 25 last sets out the award of the Albert Medal to "Miss Alice Batt, Voluntary Aid Detachment," and the services for which it was granted, which are described in our issue of May 3, p. 112. Inquiry at the Headquarters of the Voluntary Aid Detachments elicits the fact that Miss Alice Batt is a V.A.D. and not a trained nurse.—ED. THE HOSPITAL.]

